# Decrease of Plasma Platelet-Activating Factor Acetylhydrolase Activity in Lipopolysaccharide Induced Mongolian Gerbil Sepsis Model

**DOI:** 10.1371/journal.pone.0009190

**Published:** 2010-02-12

**Authors:** Junwei Yang, Jing Xu, Xiaoying Chen, Yixuan Zhang, Xucheng Jiang, Xiaokui Guo, Guoping Zhao

**Affiliations:** 1 Department of Microbiology and Li Ka Shing Institute of Health Sciences, Prince of Wales Hospital, The Chinese University of Hong Kong, Hong Kong SAR, China; 2 Shanghai Institute of Health Sciences, Shanghai, China; 3 Department of Medical Microbiology and Parasitology, Shanghai Jiaotong University School of Medicine, Shanghai, China; 4 Department of Pathology, Shanghai Jiaotong University School of Medicine, Shanghai, China; 5 School of Life Science and Biopharmaceutics, Shenyang Pharmaceutical University, Shenyang, China; 6 Laboratory of Synthetic Biology, Institute of Plant Physiology and Ecology, Shanghai Institutes for Biological Sciences, Chinese Academy of Sciences, Shanghai, China; 7 Shanghai-MOST Key Laboratory for Health and Disease Genomics, Chinese National Human Genome Center at Shanghai, Shanghai, China; Charité-Universitätsmedizin Berlin, Germany

## Abstract

Platelet-activating factor (PAF) plays an important role in the pathogenesis of sepsis, and the level of plasma PAF acetylhydrolase (pPAF-AH), which inactivates PAF, decreases in sepsis patients except for the sepsis caused by severe leptospirosis. Usually, increase of pPAF-AH activity was observed in lipopolysaccharide (LPS)-induced Syrian hamster and rat sepsis models, while contradictory effects were reported for mouse model in different studies. Here, we demonstrated the *in vivo* effects of LPS upon the change of pPAF-AH activity in C57BL/6 mice and Mongolian gerbils. After LPS-treatment, the clinical manifestations of Mongolian gerbil model were apparently similar to that of C57BL/6 mouse sepsis model. The pPAF-AH activity increased in C57BL/6 mice after LPS induction, but decreased in Mongolian gerbils, which was similar to that of the human sepsis. It thus suggests that among the LPS-induced rodent sepsis models, only Mongolian gerbil could be used for the study of pPAF-AH related to the pathogenesis of human sepsis. Proper application of this model might enable people to clarify the underline mechanism accounted for the contradictory results between the phase II and phase III clinical trials for the administration of recombinant human pPAF-AH in the sepsis therapy.

## Introduction

Platelet-activating factor (1-O-alkyl-2-acetyl-sn-glycero-3-phosphocholine, PAF), a potent proinflammatory phospholipid mediator, has remarkably diverse biological effects in diseases [1], including sepsis, which arises through body's inflammation response to infection and is a leading cause of death and disability for patients in an intensive care unit [2]. PAF synthesis is up-regulated in response to bacterial endotoxins both *in vivo* and *in vitro* [3], [4]. Although it was recently reported that PAF may protect mice against lipopolysaccharide (LPS)-mediated sepsis [5], many studies indicated that increased concentrations of PAF may contribute to the deleterious effects of systemic inflammation in the pathogenesis of severe sepsis [2].

The inactivation of PAF is mediated by PAF acetylhydrolase (PAF-AH), a calcium-independent phospholipases A2 with specificity for hydrolysis of this lipid mediator [6]. The plasma form of PAF-AH (pPAF-AH) is a secreted protein in the blood that serves to inactivate PAF and PAF-like phospholipids [6]. This enzyme accounts for all of the PAF-inhibitory activity found in human serum, limiting the normal serum half-life of PAF to only a few minutes [1], [7]. Except for the sepsis caused by severe leptospirosis [8], the activity of pPAF-AH is diminished in human sepsis [7], [9], [10], [11] as a consequence of endotoxin and cytokine-induced reduction of the pPAF-AH encoding gene transcription and possible inactivation by oxidant injury [11], [12].

A potential therapeutic strategy for sepsis is to facilitate the inactivation of PAF with the supplement of pPAF-AH. The results of the clinical trials of recombinant human pPAF-AH in patients with severe sepsis were controversial (Table 1). In 2006, Gomes *et al*. reported that the administration of exogenous recombinant pPAF-AH reduced mortality and inflammatory injury relevant to the clinical syndrome (Table 1), based on the depressed pPAF-AH activity in C57BL/6 and Swiss mouse models induced by LPS or cecal ligation and puncture (CLP) (Table 2). However, this result is partly in contradictory to the previous studies in rodents challenged with LPS, which showed an increase of pPAF-AH activity in Syrian hamsters, rats, and C57BL/6 mice (Table 2). Therefore, it would be important to clarify the response of pPAF-AH against LPS-treatment in mice, and/or explore alternative animals suitable for simulating the role of pPAF-AH in human sepsis.

**Table 1:** Therapeutic effects of the administration of recombinant human pPAF-AH in human sepsis patients and mouse models.

	Status	Year	Effect	Reference
Human	Phase IIa	1999	Improvements in oxygenation and multiple organ dysfunction	[20]
	Phase IIb	2003	Striking survival advantage and other positive effects	[21]
	Phase III	2004	No effect on decreasing mortality	[22]
Mouse	LPS/CLP model	2006	Reduction of inflammatory injury and mortality	[9]

Mouse represented the C57BL/6 mouse.

**Table 2:** Response of pPAF-AH in LPS and CLP sepsis models.

	Species	Strain	Change of pPAF-AH activity	Reference
LPS model	Hamster	Syrian hamster	↑	[14]
	Rat	Sprague-Dawley	↑	[14], [15], [16], [17]
	Mouse	C57BL/6	↑	[14], this work
		C57BL/6 and Swiss	↓	[9]
	Gerbil	Mongolian gerbil	↓	This work
CLP model	Mouse	C57BL/6 and Swiss	↓	[9]

Previously, we found that Mongolian gerbil has its normal pPAF-AH level similar to that of human, and the patterns of the change of PAF-AH level in serum during the course of severe leptospirosis in gerbil model are similar to that of severe leptospirosis patients, including the levels of elevation [8]. These findings were consistent with the fact that LPS of *Leptospira interrogans* is much less virulent than that of *Escherichia coli*, and has little effect on the sepsis caused by leptospirosis [13]. Therefore, among experimental rodents and rabbits, gerbil is likely to be a good candidate to develop an animal model to mimic the role of pPAF-AH in human diseases, particularly, the LPS-induced sepsis [8]. In this study, we examined the *in vivo* effects of LPS on pPAF-AH activity in C57BL/6 mice and gerbils.

## Materials and Methods

### Ethics Statement

All animals were handled in strict accordance with good animal practice as defined by the relevant local animal welfare bodies, and all animal work was approved by the Animal Research Committee of the Chinese National Human Genome Center at Shanghai.

### Animal Study

Male C57BL/6 mice (Shanghai Laboratory Animal Center, China), one month of age (18 to 22 g), and male gerbils (Zhejiang Laboratory Animal Center, China), two months of age (45 to 60 g), were given a standard laboratory diet and water ad libitum and housed under controlled environmental conditions. LPS from *Escherichia coli* (serotype 0111:B4) was purchased from Sigma Chemical Company and was freshly diluted to desired concentrations in pyrogen-free 0.9% saline. After a minimum 3-day acclimation period, animals were intraperitoneally injected with either saline (control) or LPS (3 or 5 mg/kg body weight).

### pPAF-AH Activity Assay

pPAF-AH activity was determined by using a commercially available assay kit (Cayman Chemical) according to manufacturer's instructions. The assay uses 2-thio-PAF, which serves as a substrate for pPAF-AH. On hydrolysis of the acetyl thioester bond by pPAF-AH, free thiols are detected using 5, 5′-dithio-bis-(2-nitrobenzoic acid) (DTNB, Ellman's reagent). The absorbance is read at 405 nm over a period of time using an ELISA plate reader.

#### Statistics

Data were analyzed with Graph-Pad Prism, version 2.0 (GraphPad Software). Data were presented as mean values ± SEM. Statistical analyses were performed using one way analysis of variance (ANOVA).

## Results and Discussion

Compared to the control group (0 mg/kg body weight, saline only), the C57BL/6 mice and gerbils with LPS-treatment (3 or 5 mg/kg body weight) appeared acutely ill and displayed signs of lethargy, and then they were euthanized while the animals appeared moribund after LPS-treatment (Table 3). Most of the animals died after LPS injection, and autopsy showed the volume increase of spleen in all the animals of both C57BL/6 mice and gerbils (Table 3). Therefore, the clinical manifestations of LPS-induced gerbil model were apparently similar to that of C57BL/6 mouse sepsis model [9].

**Table 3:** Clinical manifestations of C57BL/6 mice and gerbils induced by LPS.

	Dose (mg/kg)	Clinical observation	Death/total	Time to death (hr)
Mouse	0^a^	Normal	0/10	ND
	3	Lethargy, diarrhea and spleen volume increase	10/10	36-45
	5	Lethargy, diarrhea and spleen volume increase	10/10	15-26
Gerbil	0^a^	Normal	0/10	ND
	3	Lethargy, diarrhea and spleen volume increase	9/10	36-49
	5	Lethargy, diarrhea and spleen volume increase	10/10	13-27

Mouse represented the C57BL/6 mouse.

a Control, intraperitoneally injected with saline only.

ND, no animal death determined.

**Figure 1:**
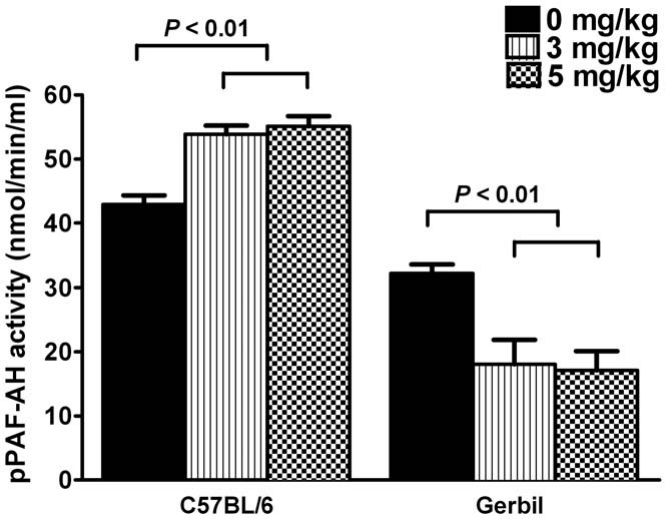
Effects of LPS on pPAF-AH activity in C57BL/6 mice and gerbils. Data were presented as means ± SEM; n = 10 for each dose group.

Blood was collected by cardiac puncture and pPAF-AH activity was measured. We found that both 3 and 5 mg/kg body weight LPS induced the elevation of the pPAF-AH activity in C57BL/6 mice, and the elevated levels were similar in these two dose group (Figure 1). In contrast, after LPS-treatment (3 or 5 mg/kg body weight), the pPAF-AH activity of gerbils decreased compared to that of the control, and the decreased levels were similar in the doses of 3 and 5 mg/kg body weight (Figure 1).

Our result showed that LPS caused the elevation of pPAF-AH activity in C57BL/6 mice (Figure 1), which was the same with that reported by Memon *et al*. [14] (Table 2), but different from the study of Gomes *et al*. [9] (Table 2). Although the reason for the different effects of LPS in C57BL/6 mice was yet to be elucidated, the present study, together with the studies in Syrian hamsters, rats, and C57BL/6 mice challenged by LPS [14], [15], [16], [17] (Table 2), showed that, among rodent species, only the gerbil demonstrated the decrease of pPAF-AH activity under the exposure of LPS (Figure 1), which was similar to the response of pPAF-AH measured in sepsis patients [7], [9], [10], [11]. Therefore, the LPS-induced sepsis in gerbil could be used to study the pharmacological effect of recombinant pPAF-AH in sepsis, and may provide pre-clinical evaluation of pPAF-AH in sepsis for the additional clinical trials in the future.

LPS is a relatively pure compound that can be stably stored in lyophilized form. Therefore, the LPS model is much easier to be established than the surgical CLP model, which is the “gold standard” in sepsis research [18]. However, the LPS model is known to be different from the sepsis in human and CLP model with respect to the profile of cytokine release [18], [19]. PAF is a phospholipid cytokine implicated in a wide range of biological and pathologic responses [1], and thus, the different responses of pPAF-AH, the regulator of serum PAF [6], between sepsis patients and LPS models of Syrian hamster, rat and mouse (Table 2 and Figure 1) might be accounted by the differences in the profile of cytokine release. The similar responses of pPAF-AH between sepsis patients and gerbil LPS model (Figure 1), as well as between the severe leptospirosis patients and the gerbil leptospirosis model [8], may implicate the similar response of cytokine release between human and gerbil. This possible underline mechanism should be further tested in order to fully characterize this novel model, which is potentially advantageous in mimicking the cytokine response in sepsis.
